# Challenges oncologists face when caring for hispanics living in puerto rico with colorectal cancer and multiple chronic conditions

**DOI:** 10.1186/s12885-025-14271-0

**Published:** 2025-05-20

**Authors:** Maira A. Castañeda-Avila, Daniela Latoni-Guillermety, Meagan Sabatino, Karen J. Ortiz-Ortiz, Kate L. Lapane

**Affiliations:** 1 Division of Epidemiology, Department of Population and Quantitative Health Sciences, University of Massachusetts Chan Medical School, 55 Lake Ave North, Worcester, MA 01655 USA; 2 Duquesne University, Pittsburgh, PA USA; 3 Cancer Control and Population Sciences Program, The University of Puerto Rico Comprehensive Cancer Center, San Juan, Puerto Rico; 4 Department of Health Services Administration, Graduate School of Public Health, Medical Sciences Campus, University of Puerto Rico, San Juan, Puerto Rico

## Abstract

**Background:**

Colorectal cancer (CRC) is the leading cause of cancer-related death in Puerto Rico, posing significant challenges for patients with multiple chronic conditions (MCC). This qualitative study aimed to explore oncologists’ perspectives regarding the care of patients with CRC and MCC in Puerto Rico.

**Methods:**

We conducted semi-structured interviews in Spanish with nine oncologists providing care for patients with CRC in Puerto Rico. We reached data saturation. We performed thematic analysis to identify key patterns and themes within the interview data. The coding scheme evolved through team discussions, with discrepancies addressed for consistency. Quotes were translated from Spanish to English.

**Results:**

Five key themes were: (1) social determinants of health, (2) diagnosis pathways, (3) factors influencing treatment decisions, (4) survivorship and end-of-life care, and (5) care coordination and communication. Oncologists treating patients with CRC and MCC identified the lack of a social support network as a notable care coordination challenge. The health insurance system’s pre-authorization requirements for procedures and treatments further complicated care delivery, particularly for older adults, who faced challenges navigating these administrative processes without sufficient support. A lack of transportation and local specialized care services was a noted barrier to comprehensive patient care. Communication between patients, physician and caregivers proved challenging when multiple physicians and procedures were involved with patient’s care, often requiring patients to schedule appointments with different specialists themselves. Inter-provider communication primarily relied on phone calls or notes sent with the patient.

**Conclusions:**

Oncologists caring for Hispanic older adults with CRC and MCC encounter complex challenges influenced by unmet social needs and the presence of comorbidities. Tailored approaches, culturally sensitive care, and improved coordination among physicians are vital to enhance the quality of care for this patient population.

**Supplementary Information:**

The online version contains supplementary material available at 10.1186/s12885-025-14271-0.

## Introduction

Colorectal cancer (CRC) is the second leading cause of cancer-related death among Hispanics in the United States [1]. Despite being more commonly diagnosed in individuals over 50 years old, a concerning trend indicates an increasing incidence of CRC among young adults, often accompanied by multiple chronic conditions (MCCs) [2, 3]. Managing CRC becomes even more complex for patients with coexisting MCCs, such as hypertension, diabetes, and depression, among others [4]. Consequently, improving cancer treatment outcomes for patients with CRC and MCC has emerged as a top priority, particularly considering the aging population and the escalating burden of CRC-related mortality and morbidity in Puerto Rico [5].

The Hispanic or Latino population is the largest and fastest-growing ethnic group in the United States, with Puerto Ricans forming the second-largest Hispanic population residing in the country [6]. Over the past decade, Puerto Rico has undergone significant demographic shifts due to a variety of factors, including the outmigration of young adults and individuals displaced by Hurricane Maria in 2017 [7–9]. As a result, the proportion of residents aged ≥ 65 years has risen from 15% in 2010 to 21% in 2019, with projections indicating continued growth [8, 9].

These demographic changes have exposed people in Puerto Rico to health disparities stemming from complex historical, cultural, social, and political factors [10]. Accessing healthcare services and resources has become economically challenging, with nearly half of the population experiencing high poverty rates [11]. Budget cuts in education and healthcare have further exacerbated these disparities [12]. Moreover, Puerto Rico’s limited political autonomy coupled with its geographical location has made its population vulnerable to natural disasters like hurricanes, further challenging Puerto Ricans’ well-being [13].

Taken together, these factors highlight a critical knowledge gap: insufficient research identifying the experiences of oncologists providing care for Hispanic older adults with CRC and MCC in Puerto Rico. To better understand these challenges and make recommendations for change, we conducted semi-structured interviews with oncologists who treat patients with CRC and MCC in Puerto Rico. By delving into their perspectives and experiences, we aimed to understand the existing obstacles and identify priorities for systemic intervention. Through this approach, we seek to offer tangible recommendations to improve care practices and outcomes for this underserved population.

## Methods

### Study design

We conducted individual semi-structured interviews with oncologists in Puerto Rico who treat patients with CRC to gain insights into the experiences, unique factors, and challenges specific to Puerto Rico’s healthcare system and cultural environment that may affect managing patients with CRC and MCC. We conducted all interviews via phone calls or Zoom and recorded them.

### Study participants and settings

We identified oncologists through the Puerto Rico Central Cancer Registry, which provided a list of practicing oncologists in Puerto Rico. Participants included oncologists currently practicing in Puerto Rico and who self-reported having experience caring for patients with CRC. Due to our study design, we excluded oncologists who were unable to commit to an interview lasting approximately 60 min or were unwilling to be audio recorded. We initially contacted 84 oncologists; of whom we heard from 23 (9 who participate and 14 who were not interested). The remaining 61 oncologists did not respond to our invitation. We reached data saturation.

We contacted oncologists, directly or through their administrative staff and provided detailed information about the study. Interested participants then coordinated specific dates and times for the interviews. Each oncologist participant could choose to have an in-person, Zoom, or telephone interview to accommodate individual preferences. MC (a female postdoctoral associate at UMass Chan at the time of the interviews) conducted the interviews, while DL (a female research assistant) assisted by taking notes, ensuring the recordings were working properly, and asking additional questions if needed. All oncologists had their interviews over Zoom or by phone. Audio recordings were collected regardless of the interview format. Before proceeding with the interviews, participants received a REDCap survey via email, which contained screening questions to confirm eligibility and collect demographic information. If a participant did not complete the survey before the interview, we verbally completed it with the participant at the beginning of the interview. Informed verbal consent was obtained from each participant before starting the study. We informed them that their participation was voluntary and that they had the right to withdraw at any time without facing any adverse consequences.

### Interview content

During interviews, we asked participants a set of open-ended questions we developed to address our study goals (Supplementary file 1). The interview guide was developed based on the cancer care continuum, which encompasses a comprehensive spectrum of care, ranging from prevention and early detection to treatment, survivorship, and end-of-life care [14]. A crucial element of effective cancer care is care coordination, which entails seamlessly integrating and managing healthcare services across various physicians and settings [15, 16]. This approach ensures that patients receive timely and appropriate care, thereby reducing gaps and inefficiencies in the healthcare system [17].

Furthermore, we acknowledged the importance of addressing social determinants of health (SDoH) in cancer care. We included open-ended questions to gather information about how oncologists perceive these factors and their experiences with them. This approach enabled us to actively recognize the significance of SDoH. Factors like income, education, housing, and access to healthcare significantly influence a person’s cancer risk, their ability to seek timely diagnosis and treatment, and their overall health outcomes [18]. Recognizing and addressing these SDoH within the cancer care continuum is vital for promoting equitable access to quality care and improving patient outcomes. We collected data on oncologist’ sociodemographic characteristics, including gender, race/ethnicity, years in practice, rural/urban location of their practice, health region, and type of practice (e.g., private, group or hospital).

### Thematic analysis

We conducted semi-structured interviews in Spanish between June and October 2022. The principal investigator (MC) conducted the interviews with assistance from co-author DL. The research team collected information from oncologists until saturation of concepts was reached. We conducted thematic analyses to evaluate the interview data and organized the coded transcript data using NVivo (QSR International https://www.qsrinternational.com/nvivo/nvivo-products). DL carried out the transcription and de-identification of all interviews. The coding process started with an unstructured read of the transcripts to familiarize ourselves with the data. Following the initial review, we generated a preliminary list of codes [19, 20]. The primary investigator (MC) developed this preliminary coding scheme after reviewing two transcripts, which DL and MS then reviewed. The research team identified interview themes thereafter. Throughout the coding process, the research team met to discuss emerging themes and to update the coding scheme as needed. DL and MC applied the coding scheme to a subset of transcripts. We met to discuss the coding, create new codes as necessary, and refine the code definitions. Following that, DL and MC applied the codes to all interviews. We logged analytic decisions and addressed disagreements during team meetings to refine code definitions and ensure consistency. Finally, DL and MC translated illustrative quotes from Spanish to English.

## Results

We interviewed nine oncologists who provide care for patients diagnosed with CRC in Puerto Rico. A majority of oncologist were female, years of practice included 22.2% in training or residence and 55.6% with less than 10 years of practice. The oncologists were roughly evenly split in terms of urban vs. rural setting. A mix of regions were represented, and all but one interviewee practiced in a private or group setting (Table 1).

**Table 1:** Self-reported characteristics of oncologists who care for patients diagnosed with colorectal cancer in Puerto Rico

Characteristics	*n*	%
**Gender**		
Female	6	66.7
Male	3	33.3
**Years in practice**		
Training/resident	2	22.2
<10 years	5	55.6
≥ 10 years	2	22.2
**Location**		
Rural	5	55.6
Urban	3	33.3
Both	1	11.1
**Health region**		
Northeast	4	44.4
Southeast	2	22.2
West	2	22.2
Central	1	11.1
**Type of practice**		
Private	4	44.4
Group	4	44.4
Hospital	1	11.1

### CRC patient characteristics

According to the interviewees, the gender distribution of CRC patients varied, with slightly more men diagnosed with CRC. On average, physicians typically saw 50–100 CRC patients per year; however, patient volume varied significantly, with some physicians seeing three to four patients per week, while others saw two to three per month.

Oncologists reported that nearly all their CRC patients had MCCs, with diabetes most commonly observed among their patients. The most frequently occurring dyad of chronic conditions was diabetes and hypertension, while the most common triad of conditions was the metabolic syndrome, comprising diabetes, hypertension, and obesity. Other conditions mentioned included high cholesterol, hypothyroidism, cardiovascular diseases, and chronic kidney disease. Arthritis, osteopenia, and osteoporosis were observed more frequently in older individuals due to their age.

Oncologists reported that patients with CRC in Puerto Rico mainly consisted of Hispanics between their mid-30s and 80 years of age. While most CRC patients were adults 65 years or older, there was a noticeable increase in younger patients in their 30s and 40s being diagnosed with CRC more frequently, often having genetic mutations or preexisting conditions.

**Fig. 1:**
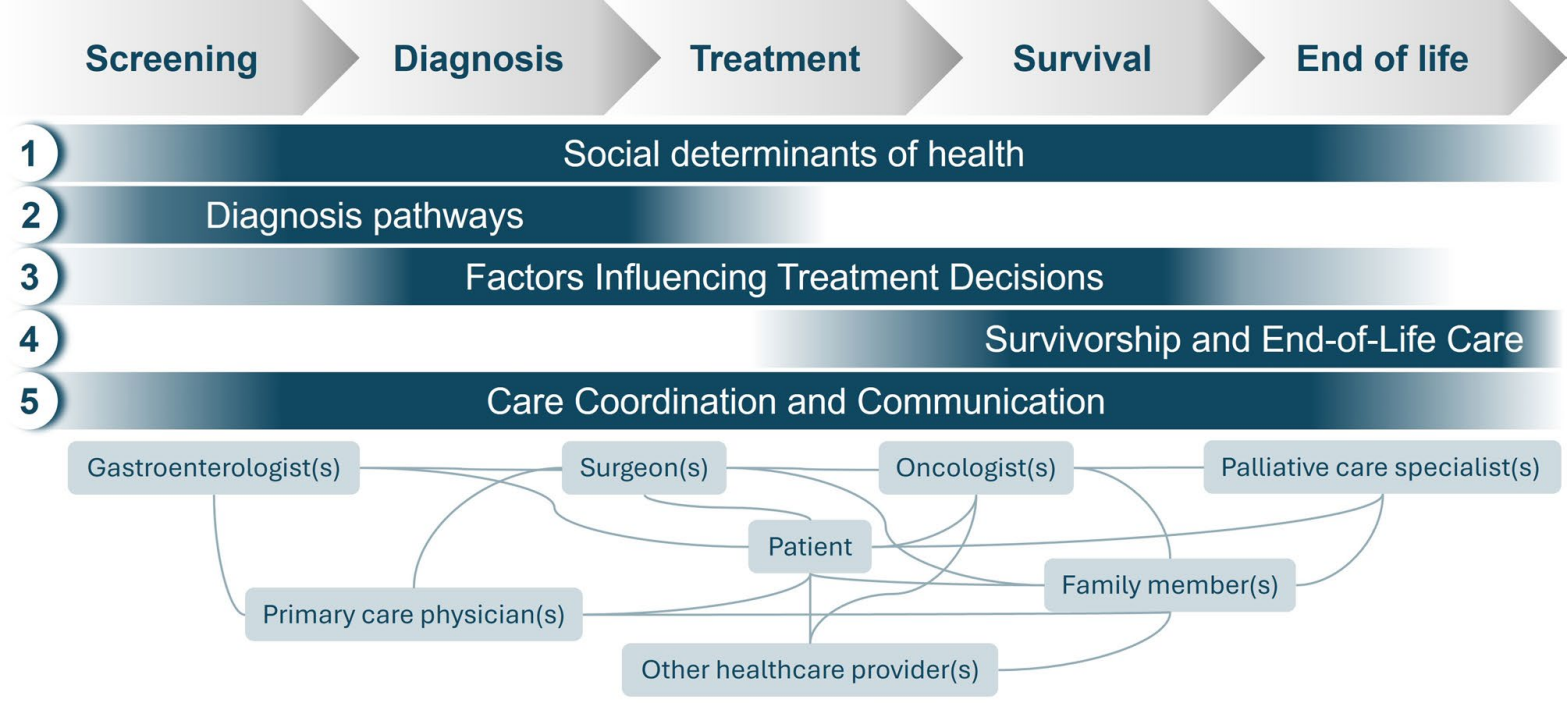
Oncologists’ interviews yielded overarching themes that span the cancer care continuum for patients with colorectal cancer and multiple chronic conditions in Puerto Rico

**Fig. 2:**
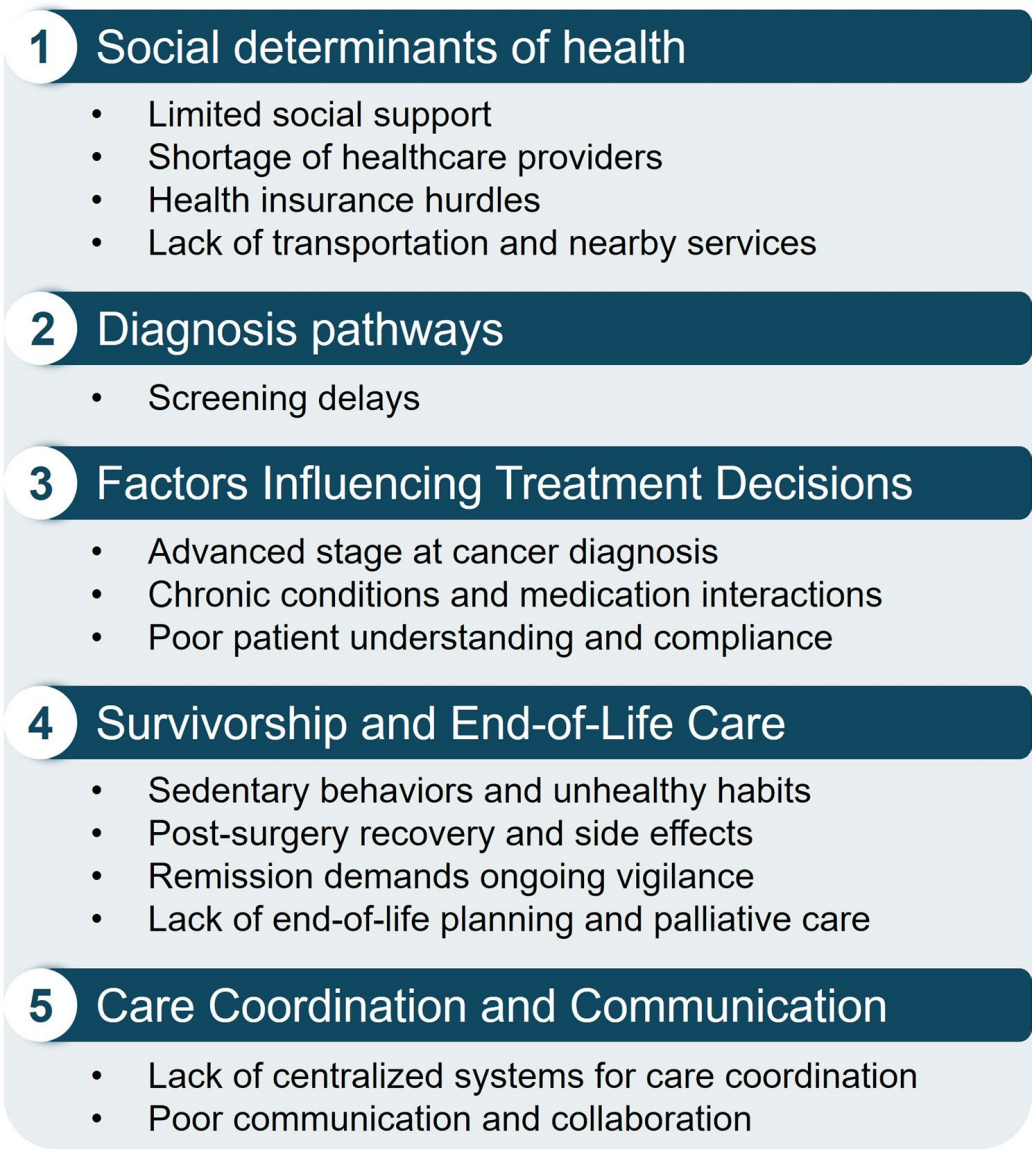
Takeaway issues across study themes

We identified five key themes: (1) social determinants of health, (2) diagnosis pathways, (3) factors influencing treatment decisions, (4) survivorship and end-of-life care, and (5) care coordination and communication (Figs. 1 and 2).

### Social determinants of health

#### Impact of limited social support

One challenge identified by physicians was the lack of social support among older patients. Many of these patients attend appointments and receive care on their own or with their partner who often was an older adult, while their family members live far away.

*“In terms of patient support and the support network*,* it’s really challenging. It’s one of the biggest frustrations that I can say I’ve experienced in the time I’ve been in this field*,* you know? Because it’s something that puts oneself in the patient’s shoes*,* and you think*,* “Wow*,* I wish I had people with me in this process.” As a physician*,* one tries*,* and I think this is the part that works the most. It’s about guiding the family on what’s happening and the seriousness of the situation to create a support network for the patient. But the reality is that sometimes one gets so busy that it becomes a bit difficult to establish or gather everyone together*,* for everyone to come and talk to you. That part is a bit difficult.”* Female, training/resident, rural.

#### Access to healthcare

Oncologists stressed the scarcity of specialized doctors (e.g., gastroenterologists, colorectal surgeons) in Puerto Rico, leading to delays in diagnosis, medical appointments, referrals, and tests. Additionally, oncologists located in rural areas mentioned insufficient psychological and emotional support resources for patients, particularly in non-metropolitan areas. Oncologists expressed heavy workloads and multiple responsibilities, including managing medications and other diseases, which ultimately contribute to burnout and may compromise the overall quality of patient care. The scarcity of specialized doctors not only hinders timely access to crucial medical services but also exacerbates the burden on existing healthcare professionals.

*“Usually*,* you know*,* everyone comes here. I’m going to be real with you. There aren’t many doctors*,* and I believe it’s going to become a crisis*,* especially after COVID. Many of my colleagues have retired*,* fallen ill*,* or even passed away for various reasons*,* and there’s a shortage of doctors. In the United States*,* I know it is common within internal medicine to specialize and follow up with cancer patients. Here*,* we don’t have that*,* and not many of my primary care doctors do it. I feel comfortable doing it because sometimes things slip through the cracks*,* you know? They see things*,* obvious red flags*,* and they miss them. So*,* I’m trying to follow all my patients for the recommended five years*,* but to be honest*,* we can’t keep up anymore. We can’t keep up because there are so many patients…”* Female, nine years in practice, rural.

#### Issues with health insurance

Oncologists expressed challenges involved with healthcare insurance, particularly related to authorization for medical procedures, delays in receiving tests and treatments due to difficulty getting care plans authorized, and limited treatment options and access to beneficial medications due to a lack of coverage. The oncologists highlighted that the pre-authorization process is time-consuming and confusing, especially for older patients. Frustrations arose when insurance did not cover treatment options or certain medications, hindering optimal patient care. One oncologist mentioned that their office has a patient coordinator to address these challenges, helping patients navigate the steps and providing assistance when patients miss appointments.

*“We have a serious problem with insurance companies. I believe that there is a serious problem with insurance companies worldwide. Wherever the healthcare system is governed by insurance companies*,* there is a limitation in treating people*,* with others making decisions about your medical judgments*,* right? You recommend something*,* but if they understand that it’s too costly*,* they have to look for alternatives for the patient. Not only that*,* but it takes a long time to get the plan*,* the health insurance*,* to cover or approve the patient’s treatment. During this time*,* the disease progresses*,* and patients get worse. Some patients even die while waiting for approval for a medication. So sometimes*,* because of this*,* one thinks*,* “Well*,* I know they will take longer to approve this medication*,* so let’s give them a cheaper one that they will probably approve faster.” Unfortunately*,* we have certain insurance companies that are a real headache when it comes to approving medications*,* limiting the patient’s access to something that would benefit them.”* Female, one year in practice, rural.

#### Lack of transportation and nearby services

Oncologists reported that the provision of medical care in Puerto Rico is significantly hampered by challenges such as distance and a lack of transportation. These issues are commonly faced in locations where limited specialized physicians and accessibility barriers impede patient care. Notably, some Puerto Rican companies aim to alleviate transportation obstacles for patients accessing healthcare services, particularly those in the metropolitan area. However, the specific challenge in Puerto Rico lies in the necessity for patients to travel extended distances to reach specialized healthcare services including surgeons and radiation oncologists. This poses a formidable obstacle for individuals residing far from these specialized services despite the existence of transportation assistance programs. The geographical dispersion of healthcare facilities in Puerto Rico makes it difficult for patients to equitably access specialized medical care.

*“Patients here usually don’t know where to go*,* and it’s difficult because they have to move from town to town to find a surgeon*,* to find a radiation oncologist*,* etc. Sometimes they live far away […] Personally*,* I have my own group of professionals that I work with*,* but sometimes the patient doesn’t want to drive an hour to see that surgeon. So*,* I tell them that they have to find another option.”* Female, four years in practice, urban.

### Diagnosis pathways

Oncologists report using diverse pathways for diagnosing CRC, including symptomatic identification, routine colonoscopies, and positive occult blood tests. They indicated that delays in colonoscopies and missed screenings were common in Puerto Rico, often leading to advanced cancer cases. Many patients arrive at their practice with advanced stages, already experiencing weight loss, metastasis, or obstructions. According to the oncologists, some younger patients with advanced cancer were initially misdiagnosed or overlooked by primary care physicians. Though most CRC patients were older adults, there was a noticeable increase in the number of younger patients being affected. This growing trend led to changes in screening guidelines, with fecal occult blood tests now recommended among adults aged 40 years and older in Puerto Rico.

*“Well*,* basically*,* I see a bit of everything*,* and the vast majority actually arrive at the hospital with noticeable symptoms*,* but they’re already bleeding. Very few cases are due to incidental colonoscopies. In other words*,* the largest number of patients are those who come in with very low hemoglobin levels that require transfusion at the hospital*,* and almost always*,* the majority already have stage 3 or stage 4 diseases.”* Female, nine years in practice, urban.

### Factors influencing treatment decisions

### Cancer stage is the most important factor

Oncologists reported that cancer stages varied among their patients. Some oncologists observed an equal distribution between early and advanced stages, while others saw predominantly advanced cases. Oncologists emphasized that the most important factor to consider when determining the best treatment for their patients was the stage at diagnosis, which they typically identified after surgery and receiving specialized test results.

*“The most important thing is the stage that the patient is in. That’s what will determine the treatment I will give. For example*,* in stage I*,* I may not necessarily have to administer treatment*,* but as we progress to higher stages*,* it becomes definite.”* Male, resident, urban.

#### Multiple chronic conditions and treatment decisions

Oncologists emphasized the need to address MCC in cancer patients. While having MCC may not directly influence treatment decisions, oncologists considered their impact on treatment and emphasized the need for close monitoring based on specific conditions. They acknowledged that appointments with patients having MCC are often longer and more complex, requiring careful evaluation of medications and treatment goals. Oncologists also stressed the importance of managing patient expectations by focusing on reducing symptoms, prolonging life, and optimizing quality of life. Furthermore, oncologists highlighted various concerns regarding medication interactions and comorbidities in cancer patients. For instance, one oncologist emphasized the importance of managing sugar levels in patients, as high sugar levels can complicate chemotherapy. Another oncologist mentioned the challenges faced by patients on warfarin, as it interacts with chemotherapy, necessitating close monitoring and adjustments. Diabetes was also a significant concern, and medications must be optimized to prevent neuropathy. Diabetes and blood pressure management were discussed in several interviews, with physicians emphasizing the importance of collaboration between oncologists and primary care physicians to ensure proper monitoring and adjustments. The use of anticoagulant medications and associated bleeding risks were also mentioned, with oncologists emphasizing the need to adjust based on laboratory results.

*“We consider whether they can tolerate the treatment or not*,* as well as any underlying conditions. For instance*,* a patient with kidney disease*,* liver disease*,* or existing diabetes-related damage in their body. We take into account the potential toxicity of the treatment based on the patient’s characteristics.”* Female, Training/resident, rural.

#### Other factors influencing treatment decisions

Oncologists indicated that other factors may influence treatment decisions, including poor patient literacy and patients’ poor physical condition. Residents and oncologists with less than 10 years of experience observed that many patients do not fully understand the importance of treatment options, potential side effects, and the need for ongoing care. This lack of awareness often leads to poor compliance with the prescribed treatment, necessitating adjustments to the treatment plan.

*“If the patient is unlikely to attend appointments*,* if hospitalization is required for treatment*,* or if the patient seems to have poor compliance*,* we adjust the treatment accordingly. If the patient is likely to miss appointments*,* then we opt for a less frequent treatment.”* Female, one year in practice, rural.

### Survivorship and end-of-life care

#### Impact of lifestyle choices

Oncologists also emphasized the impact of lifestyle choices in the care of these patients. Oncologists reported that their patients were sedentary.

*“Here in Puerto Rico*,* the majority of patients I see are who don’t exercise*,* don’t eat healthy*,* who are inactive and sedentary. That’s the challenging part. And because they’re more sedentary and engage in those habits*,* they apply the same approach to their health. You understand? If they’ve had surgery*,* they think*,* well*,* that’s it*,* it’s over*,* I’m cured. It’s more than just education; it’s a mindset.”* Female, four years in practice, urban.

#### Post-surgery recovery and side effects

Post-surgery recovery and side effects pose significant challenges in the field of oncology. Oncologists highlighted the difficulties in managing severe side effects and ensuring patient adherence to treatment regimens. Though some cases may involve early-stage diagnoses that only necessitate surgical interventions, the persistence of side effects becomes a crucial concern for medical professionals. Specifically, common side effects following surgery for cancer treatment may include pain, fatigue, nausea, and changes in bodily functions. Surgical procedures themselves can result in complications such as infections, bleeding, or wound healing issues. Additionally, patients may experience emotional and psychological challenges, including anxiety and depression, which can impact their overall well-being. Addressing and managing these side effects is vital for ensuring a successful recovery and maintaining patient compliance with the recommended treatment plan. Oncologists remained committed to following up with patients, offering ongoing support, and adjusting treatment strategies as needed to enhance the overall quality of care and patient outcomes.

*“Once a treatment begins*,* the biggest challenge is to keep patients in the treatment because*,* you know*,* even though the treatments are done for their own good*,* they have side effects. Particularly in colorectal cancer*,* most treatments are based on chemotherapy*,* which causes neuropathy*,* diarrhea*,* which are symptoms that greatly affect the patient’s daily life. It’s not just experiencing nausea or vomiting once and it’s over. No*,* it’s living with weak hands*,* weak feet*,* and bothersome diarrhea*,* you know?”* Female, one year in practice, rural.

#### Managing cancer remission

Managing cancer remission posed a unique challenge for oncologists. Oncologists highlighted the need for ongoing vigilance beyond established surveillance guidelines. Despite well-defined protocols for monitoring patients in remission, oncologists often chose to follow their patients indefinitely, scheduling regular appointments, typically between one and two times per year. The reason behind this extended follow-up lies in the unpredictable nature of cancer and the potential for recurrence. Oncologists recognized that even after successful treatment and a period of remission, cancer can return. They recognized that continuous monitoring is key in their approach. It allows them to catch any signs of recurrence or new developments early on, which is crucial for oncologists to intervene promptly and increase the chances of successful treatment. The commitment to ongoing follow-up underscores the importance of personalized and attentive care, ensuring that patients receive comprehensive support throughout their journey, even in periods of apparent recovery.

*“Even though those guidelines are established*,* and the patient is in remission*,* the guidelines state five years*,* but in private practice*,* once those five years have passed*,* there’s no need to come anymore. No*,* they continue coming at least once a year*,* and many times we do it twice a year. We evaluate them*,* physical examination*,* lab tests*,* markers*,* and that’s to ensure that they are doing well. And beyond that*,* it reassures them*,* it gives them that sense of security that I’m going to try again*,* I’m doing well for the patient and for the people who care. So yes*,* we always end up following them indefinitely until the patient decides otherwise.”* Female, four years in practice, urban/rural.

#### End-of-life planning and palliative care

Factors considered when determining appropriate care for terminally ill patients include their dependency on transfusions, their overall physical condition, and their quality of life. Oncologists indicated that hospice care was often recommended for patients who may benefit from the support and assistance provided by hospice teams. However, some oncologists continued to follow and provide care to patients who did not transition to hospice, offering palliative support and addressing specific needs until the end of life.

*“The hospital also has an End-of-Life service*,* which is activated when the patient is quite ill*,* providing the palliative support they need at the end of life. However*,* some patients may not want it. They may not want to be in hospice*,* and obviously*,* I respect that decision. In such cases*,* we try to provide them with relief for their pain*,* shortness of breath*,* and help with their nutrition*,* whatever they need at that moment. That’s how we mostly handle it here in our hospital.”* Female, nine years in practice, urban.

### Care coordination and communication

#### Coordinating patient care

Oncologists recognized their role in coordinating the care of patients with CRC. However, they indicated that patients are primarily responsible for scheduling their appointments and coordinating their care. The absence of centralized health information technology systems and comprehensive care centers further complicates navigating the healthcare system, making it harder to ensure timely and coordinated care. Oncologists mentioned that some patients are referred to the cancer center for cancer treatment for various reasons, including complex cases requiring specialized and multidisciplinary care. These referrals are often based on genetic testing results or the availability of newer medications suitable for specific mutations. Medical centers affiliated with educational institutions facilitate communication and collaboration among different specialties, maintaining good communication with referring physicians and healthcare professionals involved in the patient’s care. However, communication tends to decrease once treatment begins, unless significant changes or concerns arise.

Oncologists also emphasized the importance of involving a patient’s primary care physician in cancer care, especially for managing other chronic conditions they may have, necessitating communication with other physician, such as cardiologists, for medication adjustments. Challenges can arise due to medication interactions, particularly in regions with limited resources or smaller clinics. Additionally, oncologists often have their own groups of physicians whom they reach out to for support when needed. However, some oncologists prefer to manage all conditions independently.

*“It’s a little more difficult here than in the United States*,* where they have centers and everything is taken care of*,* with a case manager helping them navigate through it all. Here*,* it’s a bit more challenging*,* and the patient has to do it on their own. They have to find a surgeon*,* and an interventional radiologist; I can provide a referral*,* but they have to schedule the appointment. You see*,* it’s usually not done from the office unless it’s an emergency that needs immediate attention. So*,* the patient makes the calls and handles all of that*,* and usually*,* it’s the patient themselves who takes care of everything*,* unless it’s a large center or a specialized facility*,* which we don’t have here yet.”* Female, four years in practice, Urban.

#### Inter-provider communication and collaboration

Oncologists indicated that communication between physicians occurs through various methods such as phone calls, written notes, and email. Direct phone calls are preferred for immediate concerns, whereas written notes are typically used for non-urgent matters. The frequency of communication varies depending on the patient’s needs, with more intensive communication for complex cases or treatment adjustments. The most reported method of communication was through written notes with patients to share with the members of their care team.

*By phone*,* or if the patient is*,* in my opinion*,* not an emergency case*,* I write them a note*,* and they go and*,* on their next visit*,* bring me the feedback of what the doctor did or what was agreed upon with their primary physician or the subspecialist I referred them to.”* Female, nine years in practice, urban.

## Discussion

In Puerto Rico, CRC represents a significant public health concern, as it is the leading cause of cancer-related deaths in the region. The management of CRC is complicated by the presence of MCC among patients, posing challenges for oncologists to effectively coordinate patient care. In this qualitative study of oncologists in Puerto Rico, we revealed several key themes related to the care of patients with CRC and MCC. Most oncologists reported a high prevalence of MCC among their patients with CRC. Coordination and delivery of patient care were complicated by insufficient social support networks and cumbersome health insurance processes, such as prior authorization. Unmet social needs, such as the lack of transportation, were additional barriers to comprehensive care for patients with CRC and MCC. Finally, inter-provider coordination and communication proved challenging, often requiring patients to schedule their appointments and facilitate communication between their physicians without support.

Notably, oncologists reported a high prevalence of comorbidities such as diabetes, high blood pressure, cholesterol problems, and hypothyroidism among their patients with CRC. Similarly, studies in Spain, Canada, and the United States found that CRC patients had a high prevalence of MCC, with the most common comorbidities including diabetes, chronic obstructive pulmonary disease, osteoarthritis, anxiety, and congestive heart failure [21, 22, 23, 24]. Patients with both CRC and MCC typically require care from oncologists, primary care physicians, and multiple other healthcare specialists to manage their diagnoses. For effective healthcare delivery that arranges the most favorable circumstances to optimize care outcomes, it is crucial that these healthcare specialists provide well-coordinated care. Care coordination is described as deliberately organizing care activities, such as medication or symptom management, for a patient and sharing their healthcare information among all physicians involved in their care to achieve safer and more effective outcomes [25]. For patients diagnosed with cancer, coordinating activities such as cancer screening and surveillance, medication management, management of the effects of cancer and treatment, and preventative care require communication between oncologists, primary care physicians, and other specialists [26, 27]. Care coordination, including activities such as prescribing medications, is an especially important aspect of care for patients with MCC [27].

Care coordination and communication emerged as significant challenges faced by oncologists treating patients with CRC and MCC. The need to receive care from multiple physicians often requires patients to schedule appointments with different specialists and leads to increased delays and fragmented care. Care fragmentation is a significant concern in healthcare due to its potential to lead to medical errors, unnecessary visits, avoidable hospitalizations, and suboptimal care resulting from incomplete information sharing among multiple physicians [28]. A previous systematic review revealed physician’ perspectives on care coordination challenges for adults with cancer and MCC included navigating multiple treatment regimens, potential drug interactions, communication barriers, and the need for enhanced collaboration among specialists [29]. Furthermore, they identified that patients diagnosed with both CRC and MCC tend to experience worse clinical outcomes compared to patients with CRC alone, regardless of age [29]. They also found that while clinical outcomes for patients diagnosed with CRC without MCC have shown significant improvements, it remains unclear whether these advancements have been observed in patients with additional MCCs [29]. Improving communication and coordination among the multiple physicians treating patients may lead to improved care experiences and outcomes for patients with CRC and MCC [29]. Approaches that have been found to help with care coordination include colorectal multidisciplinary team meetings, defined by the National Cancer Institute as a treatment planning approach in which several doctors, each an expert in different specialties, review and discuss the patient’s medical condition and treatment options [30, 31]. Other programs include care navigation, where trained professionals assist patients in scheduling appointments, understanding treatment plans, and communicating with various healthcare providers [32].

Social determinants of health emerged as a critical factor influencing cancer care among Hispanic patients with CRC and MCC. Limited social support was identified as a care coordination challenge, and many patients underwent treatments without support networks. Previous studies have shown that caregivers, particularly spouses or partners, play critical roles in providing care and support to older adults with cancer and improve care outcomes [33, 34]. However, patients’ children and other extended family members often live far away, which limits available social support, presents difficulties with care coordination, and creates related financial burdens [35, 36, 37, 38]. The financial burden on Hispanic caregivers is also a growing concern due to travel costs and lost wages [39]. About seven million family caregivers are managing their relatives’ care remotely in the US, with this number expected to increase over time [36]. Previous studies have discussed the challenges faced by Hispanic cancer caregivers in the US, highlighting the importance of cultural values and beliefs in shaping the caregiving experience [39, 40]. To address these challenges, potential solutions include enhancing support systems through community-based programs [41, 42].

Access to healthcare services was a noted concern, driven by both a shortage of physician and the absence of specialized cancer care in some areas [43]. The oncologists noted that the health insurance system poses challenges, particularly regarding the requirement of pre-authorization for various procedures and treatments. Patients with multiple chronic conditions experienced a longer time-to-surgery [21]. In Puerto Rico, advanced stage CRC was more common in Government Health Plan patients, with Government Health Plan patients in the 50–64 and 65 + year age groups having a greater excess risk of death compared to Non-Government Health Plan patients [44]. In response to this concerns, potential solutions include expanding telemedicine services to provide greater access to specialized care, even in underserved areas [45, 46]. Streamlining the pre-authorization process and advocating for policy changes to simplify and expedite approvals can also improve patient care and reduce delays.

The oncologists reported that the diagnosis and treatment pathways for patients with CRC varied. Some patients had their CRC identified through routine screenings, such as fecal occult blood tests, while others were presented with symptoms. Regardless of how CRC was initially identified, it remains unknown if adequate follow-up colonoscopies are being performed, potentially causing delays in diagnosis and initiation of treatment. Furthermore, oncologists in our study stated that the decision-making process for treatment plans is complex, and various factors, such as cancer stage, the presence of MCC, and the patient’s ability to tolerate treatment, are considered. Previous studies have highlighted the importance of considering individual patient characteristics, tumor characteristics, and the overall health status of the patient to make treatment decisions for patients with CRC [47]. While treatment plans vary depending on the cancer stage, the presence of MCC can further complicate the decision-making process. The patient’s overall health, including their ability to withstand the side effects of treatment, is a critical consideration for oncologists. Additionally, biomarkers and genetic testing may also help guide treatment decisions, as these factors can provide valuable insights into the tumor’s molecular profile and response to specific treatments [47]. Finally, patient preferences and values play an essential role in the decision-making process, as treatment choices should align with patient goals and quality of life preferences [47].

For patients in advanced stages of CRC, end-of-life planning and palliative care are important considerations for oncologists. Early discussions around hospice care, pain management, and symptom relief are essential for ensuring a dignified and comfortable transition for these patients. A previous study in Puerto Rico found a high proportion (54.3%) of patients with gastroenterological cancer receive high-intensity care at the end-of-life, including aggressive treatments such as ER visits, hospitalizations, ICU admissions, and chemotherapy use in the 14 days before death [48]. The low utilization of hospice care (20.3%) raises concerns about inadequate symptom management and higher emotional distress at end-of-life [48]. Further research and tailored interventions are needed to improve end-of-life care for cancer patients in Puerto Rico, with a focus on increasing access to hospice care and palliative medicine specialists, and addressing cultural factors and barriers that may influence end-of-life decisions and experiences.

### Strengths and limitations

This study is not without limitations. The focus on a specific geographic location (Puerto Rico) and patient population, which may limit the transferability of the findings to other contexts beyond Puerto Rico. Different regions and diverse patient populations may encounter distinct challenges in cancer care that might not be fully captured by this study. While the study might be partially generalizable to patients with access to healthcare facilities with low resources, generalizability to patient populations with access to high resource facilities might not be applicable. Future studies should evaluate differences between low and high resource facilities. We acknowledge that including trainees is a limitation, as their responses might reflect their supervising attendings’ perspectives rather than their own prolonged clinical experience. However, no major differences between oncologists with less experience and experienced oncologists were observed. The only difference we found was that trainees did not indicate a lack of providers or delays in treatment initiation. Conversely, more experienced oncologists (> 10 years) did not mention patients’ lack of awareness/education regarding the necessary actions or steps that affect cancer care. As with any qualitative study, our results could have been influenced by selection bias, the oncologists’ recall bias, and personal biases held by the researchers. To mitigate these biases and ensure rigor within this study, we employed convenience sampling to capture diverse perspectives, used semi-structured interviews, and conducted member checking for validation. We also maintained reflexivity and adhered to qualitative research standards. These strategies enhanced the credibility and trustworthiness of our findings despite limitations. Despite these constraints, this study provides in-depth insights into the challenges faced by oncologists providing care to CRC patients in Puerto Rico. The use of semi-structured interviews allowed for a detailed exploration of the experiences and perspectives of oncologists providing care for Hispanic older adults with CRC. This study is a first step to understand the issues, facilitators, barriers, and potential solutions from the perspective of oncologists. The diverse range of viewpoints among enhance the credibility of the findings. Thematic analysis facilitated the identification of key themes and subthemes, ensuring that the results were grounded in the data and providing a comprehensive understanding of the challenges faced by healthcare physicians.

## Conclusion

The identified themes underscore the critical role of addressing social determinants of health, tailoring care approaches, and improving care coordination to enhance the quality of care for Hispanic adults with CRC and MCC in Puerto Rico. Policymakers and healthcare professionals should use these findings to inform tailored approaches that improve cancer care outcomes for Hispanic older adults with CRC and MCC in Puerto Rico.

## Electronic supplementary material

Below is the link to the electronic supplementary material.

Supplementary Material 1

## Data Availability

Enquiries about data availability should be directed to the corresponding author.
