# Supplementary material for: Challenges oncologists face when caring for hispanics living in puerto rico with colorectal cancer and multiple chronic conditions
Source: BMC Cancer. 2025 May 20;25:898. doi: 10.1186/s12885-025-14271-0 (PMC12090652; doi:10.1186/s12885-025-14271-0)
Supplement: Supplementary file 1 — Supplementary Material 1 [file 12885_2025_14271_MOESM1_ESM.docx]

**INTERVIEW PROTOCOL FOR ONCOLOGISTS**

*This document is to be used as a discussion guide during the interview session. It should not be used verbatim as a questionnaire. The questions and probes may be used differently depending on the participant feedback and level of comfort with the process. The interviewer may ask probing or follow up questions to more fully explore or clarify issues brought up on a given question or over the course of the entire interview.*

**Preparation for the interview**

**Materials: In-Person**

• Refreshments

• Recorders/Computers (2) – charged/backup batteries

• Mics (2) – backup batteries

• Fact Sheet

**Materials – Phone or Online Video**

• Phone recorder – charged/backup batteries

• E-mail to participant ahead of time: Fact Sheet

**Welcome**

**Intent:** The goal of section 1 is to greet the participant, explain the study and obtain verbal consent.

- Greeting the participant:

*“Hello, my name is _____ and I will be talking with you about your experience caring for patients with colorectal cancer and multiple chronic conditions”.*

- Thank the participant for talking the time to participate in our study:

*“Thank you very much for being a part of this study and taking the time to speak with me today”*

- Explain the study:

*“Just as a reminder, our goal is to explore healthcare providers’ perceptions and experiences caring for older Hispanic adults with colorectal cancer and multiple chronic conditions”*

- Review informed consent and obtain verbal consent
- Explain that we will be audio-recording the face to face, zoom or phone interview so we can gather more information from their stories later.

*“First, we need to complete the consent process. I’d like to record this interview if that’s ok with you. We will store the de-identified transcript on our password protected computer.”*

- Inform participant that recorded data is in no way linked to the study subject and that we will not be using their name in the recording
  - Ask participant to refrain from using names – particularly names of patients – during the recording
- Initiate recording

*“I am now turning on the recorders.”*

- - Turn on recording.
  - Begin the recording by stating the date and time.
  - Confirm permission to record the encounter.

**Interview**

***General practice***

1. Tell me a little bit about your practice in general.
   - Do you see many patients with colorectal cancer?
   - About how many over the course of a year?
   - Can you describe the profile of these patients?
   - About how many are older adults (65+ years of age)?
   - Are they in relative good health (aside from their cancer)?
   - Do most come to you with symptoms?
   - Or were they referred after a routine colonoscopy?
   - Anything else to share?
   - Have you seen changes in the types of patients you see over time? are they older? Younger? Any thoughts?
2. Can you tell me about the typical process when a patient is referred to you because of a colorectal cancer diagnosis?
   - What information do you collect on intake? (Probe: Medical history? Medications? Any thing else?)
   - Do you determine the stage of the colorectal cancer or has that been determined already when they come to you?
   - If a patient comes to you without the stage determined, tell me about your typical processes for these patients? (e.g., tests, labs, images, etc). How long does this process take?
   - What factors you take in consideration to decide the treatment plan? Does a patient’s age enter into your decision making process? What about comorbid conditions? Do some comorbid conditions influence your treatment plan more than others? (e.g., diabetes, inflammatory diseases, etc.?)
   - As part of the treatment plan, do you refer the patient to other providers? If so, who? (E.g., surgeons, radiologists, etc)
   - What factors do you take into consideration to determine that the cancer cannot be controlled? Do you talk to the patient or their relatives about palliative care? What services do you refer this patient to? (For example, pain management, psychological care, hospice, etc.)
3. In thinking about the patients you see with colorectal cancer, about what proportion are older adults 65 years of age and older? Would you say more than 75%? 50-75%? 25-50%?

***Patients with Multiple Chronic Conditions***

Now focusing on the older adults who come to you with colorectal cancer, I am interested in learning more about whether patients have multiple chronic conditions like diabetes, hypertension, depression, cardiovascular diseases, autoimmune diseases, among others.

1. Can you give a rough estimate of the percent of older adults presenting with colorectal cancer who have multiple chronic conditions in your practice? would you say more than 75%? 50-75%, 25-50%?
   - What are the most common chronic conditions these patients have?
   - What is the most common combination (dyads) of multiple chronic conditions?
2. Can you think of a particular case example of caring for an older adult with colorectal cancer and multiple chronic conditions and could you tell me a little bit about it?

- Did you manage this patient on your own?
- Who did you work with to manage their care? (Probe: if they are focused on the colorectal cancer care and just talk about surgeons, radiologists, etc; ask about other conditions mention earlier)
- Did you coordinate care with primary care providers? Other providers who may be managing their chronic diseases (e.g., cardiologists for CVD, or endocrinologists for diabetes, etc.)
- What informed your decision making?

1. Now earlier, you told me that it was typical practice to gather medical history and (whatever they told on question # 2)
   - How is this information being used in patient care process?
   - Does it affect what treatment options your recommend?
   - Does it influence any pre-operative instructions you give? (e.g., do you ask patients to stop taking any medications if surgery is recommended)
   - Does it affect any post-operative recommendations?
   - Do you do anything differently in your practice to identify patients with chronic conditions?
   - Do you tag patients with multiple chronic conditions in the medical record for identification purpose?
   - Are there some chronic conditions that concern you more than others with respect to your treatment plans for colorectal cancer? What are they? Why are they of a concern?
   - Many patients with multiple chronic conditions are also on multiple medications. Are you concerned about this in your patients with colorectal cancer? Are there any medications in particular that concern you when caring for older adults with colorectal cancer?
2. When developing their treatment plans, do you do anything differently for patients with colorectal cancer and multiple chronic conditions than for patients with colorectal cancer without multiple chronic conditions? If so, what is different? If not, can you imagine a circumstance where your regular practice may differ because of multiple chronic conditions? Tell me more about that.
3. As for patients considered at the end of life, what treatments or services do you offer them? Can you tell me more about the experience with this patients.

***Care coordination***

I would like to switch gears now to talk about care coordination.

1. I’m wondering whether it is usual practice to coordinate care for patients with colorectal cancer and multiple chronic conditions. Is that something that routinely happens?
   - If no, ideally should more coordination occur?
     1. If so, how do you think that could happen?
     2. What are the barriers to coordinated care?
   - If yes,
     1. Tell me more about how you communicate with these providers? (Probe: ever talk in person? By email? Through electronic medical records? What are the communication platforms?)
     2. How often do you communicate with these providers?
   - Do you typically reach out to let them know patient progress? To get information you need before deciding on a treatment plan?
2. What are some challenges encountered in taking care of patients with colorectal cancer and multiple chronic conditions?
   - How can this be improved?
3. Who monitors for side effects? And chemotherapy and drug interactions?
4. If a patient only required surgery as part of the treatment. Do you continue follow-up patient after the surgery? For how long? Or who is responsible for follow-up this patient?
5. If cancer is in remission who should continue providing follow-up to those patients?
6. As for patients consider at the end of life, who should continue providing follow-up to those patients?

**Closing and follow up**

We are about to end the interview. Is there anything you’d like to add about how you care for patients with colorectal cancer and multiple chronic conditions?

Do you have any questions for me?

Would you like a copy of our research findings when we are done?

Thank you for talking with me. I’m going to shut off the recorder now.

**TURN OFF RECORDER.**

I appreciate all the information and feedback you have given me.

- Thank the provider for their valuable contribution to this research.
- Immediately after the interview (after you have left the participants’ area or ended the phone interview), write down any general reflections about the interview, thoughts about the participants attitudes or reactions, or any other potentially pertinent info.
